# Impacts of twenty years of experimental warming on soil carbon, nitrogen, moisture and soil mites across alpine/subarctic tundra communities

**DOI:** 10.1038/srep44489

**Published:** 2017-03-15

**Authors:** Juha M. Alatalo, Annika K. Jägerbrand, Jaanis Juhanson, Anders Michelsen, Peter Ľuptáčik

**Affiliations:** 1 Department of Biological and Environmental Sciences, College of Arts and Sciences, Qatar University, P.O. Box 2713, Doha, Qatar; 2 Calluna AB, Hästholmsvägen 28, 131 30 Nacka, Sweden; 3 Swedish University of Agricultural Sciences, Department of Forest Mycology and Plant Pathology, P.O Box 7026, SE-75007 Uppsala, Sweden; 4 Terrestrial Ecology Section, Department of Biology, University of Copenhagen, Copenhagen, Denmark; 5 Center for Permafrost (CENPERM), University of Copenhagen, Øster Voldgade 10, DK-1350 Copenhagen K, Denmark; 6 Institute of Biology and Ecology, Faculty of Science, P. J. Šafárik University in Košice, Šrobárova 2, 041 54 Košice, Slovakia

## Abstract

High-altitude and alpine areas are predicted to experience rapid and substantial increases in future temperature, which may have serious impacts on soil carbon, nutrient and soil fauna. Here we report the impact of 20 years of experimental warming on soil properties and soil mites in three contrasting plant communities in alpine/subarctic Sweden. Long-term warming decreased juvenile oribatid mite density, but had no effect on adult oribatids density, total mite density, any major mite group or the most common species. Long-term warming also caused loss of nitrogen, carbon and moisture from the mineral soil layer in mesic meadow, but not in wet meadow or heath or from the organic soil layer. There was a significant site effect on the density of one mite species, *Oppiella neerlandica*, and all soil parameters. A significant plot-scale impact on mites suggests that small-scale heterogeneity may be important for buffering mites from global warming. The results indicated that juvenile mites may be more vulnerable to global warming than adult stages. Importantly, the results also indicated that global warming may cause carbon and nitrogen losses in alpine and tundra mineral soils and that its effects may differ at local scale.

In Arctic areas, future climate change is predicted to prolong the growing season, with the shift being more pronounced in colder mountainous parts, an effect that will most likely cause retreat of the tundra zone and changes in the tree line^1^,^2^,^3^. Global warming may also affect soil carbon storage and, as Arctic and alpine tundra contains almost half of all global soil carbon, there is a need for studies on the potential impact of global warming on the carbon balance^4^,^5^,^6^. Nutrient mineralisation in soils is predicted to increase as a response to future warming^7^,^8^. A recent study investigating two decades of experimental warming observed no impact on soil carbon storage^4^. However, long-term fertilisation has been shown to cause a loss of soil carbon^9^. A growing number of studies are examining the potential impact of global warming on plant communities. The results obtained to date indicate that warming may cause an increase in biomass^4^, shifts in the dominance structure of plant communities and an increase in shrubs in the Arctic^10^,^11^. However, fewer studies have investigated soil fauna, which play a vital role in carbon cycling^12^. This applies even for polar and alpine regions, where global warming is predicted to be both rapid and severe. Furthermore, most existing studies on soil arthropods in polar and alpine regions are short-term^13^,^14^,^15^ and to our knowledge there are only two long-term experimental warming studies, one with 11 and 12 years of warming^16^ and one with 16 years of warming^17^.

Soil mites play a vital role in several soil processes and are therefore important for ecosystem functioning and carbon cycling^12^,^18^. They include saprophagous mites that contribute to decomposition processes (e.g. Oribatida) and predatory mites (e.g. Gamasina). In polar and alpine regions, where there is a lack of larger invertebrates, the relative importance of mites is even greater for decomposition-related soil processes^19^. In higher diversity systems, functional diversity seems to be more important than species richness^20^,^21^, while in low diversity systems the number of species is more important^22^. This may be because there are fewer species to take over new functional roles if other species are lost^22^. The temperature and humidity of soils are important limiting factors for the distribution and diversity of mites^23^. However, mites in polar regions are well-adapted to the cold environment^23^, and are often able to remain dormant for most of the year and become active during the short polar summer^24^. Importantly, experiments have shown that there is a positive relationship between soil biota and carbon cycling in low diversity systems, but less so in high diversity systems^12^.

Previous studies on the effects of experimental climate warming on microarthropods report contrasting responses, from increased mite densities in polar ecosystems^13^,^14^,^16^ to no response^25^,^26^. These responses are suggested to be both habitat- and group-specific^27^. For example, in one study oribatid mite populations were shown to increase and mesostigmatid mites to decrease in response to warming in a glade and fell field, but not in a heath^16^. Negative effects on microarthropods have mainly been reported for groups sensitive to low soil moisture, especially in ecosystems where soil moisture may already be a limiting factor^27^. Mites are generally more tolerant to high temperatures than Collembola^28^,^29^, but water stress is likely to affect soft-bodied prostigmatid mites and nymphs of other mites^30^,^31^. In general, unless associated with decreased soil moisture, increased warming in the polar regions is predicted to increase soil invertebrate numbers due to microbial communities and plant communities increasing in productivity and complexity^27^. However, a recent long-term study found no impact of experimental warming on Collembola at sub-arctic/alpine sites^32^. In the present study, we examined the impact of 19 and 21 years of experimental warming, field site and plot scale on soil parameters (soil carbon (C), nitrogen (N), C/N ratio, moisture and pH in the organic and mineral soil layers), total mite density, juvenile and adult stages of mites, density of major mite groups and the most common species of oribatid mites in three contrasting alpine/subarctic plant communities.

## Methods

### Study area

The three experimental sites were located at the Latnjajaure field station in northern Sweden, at 1000 m elevation in the Latnjavagge valley (68°21′N, 18°29′E) near Abisko. The sites, wet meadow, mesic meadow and dry heath, were all within 300 m from each other, with elevation differing by ~20 m. The climate in the area can be classified as subarctic, with cool summers, relatively mild, snow-rich winters and snow cover for most of the year. Mean annual temperature ranges from −1 to −3 °C and total annual precipitation from 600 to 1100 mm. The Latnjavagge valley is highly diverse in terms of physical conditions, ranging from dry, nutrient-poor and acidic to wet and base-rich, variations reflected in its plant communities^33^,^34^. The mesic meadow community is dominated by *Carex vaginata, C. bigelowii, Festuca ovina, Salix reticulata, S. polaris, Cassiope tetragona, Polygonum viviparum* and *Thalictrum alpinum*^33^,^34^. The more sparsely vegetated poor heath community is dominated by *Betula nana, S. herbacea* and *Calamagrostis lapponica*^32^,^33^. The well-developed wet meadow is dominated by *Parnassia palustris, Petasites frigidum, P. viviparum, Ranunculus acris, S. lanata, S. polaris*, and *T. alpinum*^35^.

### Experimental design

Warming was induced using open top chambers (OTCs), which increase the temperature by 1.5–3 °C compared with control plots with ambient temperature^33^. We chose three contrasting types of habitats to obtain a range of soil moisture levels from wet (wet meadow), to intermediate (mesic meadow) and dry (poor heath) when assessing the effects of long-term warming on vegetation and fauna. In the wet meadow community, 10 plots with homogeneous vegetation cover were marked out in 1993 and half were assigned to OTC and half to control plots in a pairwise design. In the present study we sampled four OTCs and their control plots in the wet meadow to give equal sample size to the mesic meadow and poor heath sites. In the mesic meadow and heath communities, eight plots (1 m × 1 m) were randomly assigned to treatments in 1995, half to OTCs and half to controls. The OTCs were left on plots with warming treatments year-round at all three sites.

### Sampling and analyses

At the peak of the growing season (26 July 2013), we extracted three soil cores per plot from four plots with OTCs and four control plots in each of the three plant communities. At sampling, the wet meadow had experienced 21 years of experimental warming and the mesic meadow and poor heath 19 years. The samples comprised soil cores 3.6 cm in diameter (10 cm^2^ in area) and with a maximum depth of 7–12 cm (depending on the soil depth). As the plots are part of long-term experiments on plant communities, we sought to avoid large-scale destructive sampling that would impede future research. The samples were stored in plastic bags in coolboxes until extraction, which was performed within 5 days of field sampling using a modified high-gradient extraction apparatus^36^ for 7 days. In order to study the impact on different functional groups (predators, saprophages), soil mite specimens were identified using morphological methods to species (oribatid adult individuals only) and group level as: Prostigmata (predators/saprophages), Astigmata (saprophages), Gamasina (predators), Uropodina (saprophages) and Oribatida (saprophages). Determination to order, suborder or cohort level was performed in a small glass bowl using a stereomicroscope (Olympus SZ61) according to Krantz & Walter^37^. Oribatid mites were temporarily mounted onto microscope slides with lactic acid for lightening. Species determination was conducted using a light microscope (Leica DM1000) and an identification key for mites^37^,^38^,^39^,^40^,^41^,^42^,^43^.

### Soil chemical analyses

Soil moisture was determined by oven-drying soil at 105 °C to constant weight. Soil pH was measured in soil:water suspension (1:5 ratio, w-v) with a digital pH meter. Subsamples of soil material were weighed and packed into tin capsules, with sample size adjusted between 5 and 30 mg according to the N and C concentration present. The concentrations of soil elements were analysed at the Department of Biology, University of Copenhagen, with an Isoprime isotope ratio mass spectrometer using continuous flow (Isoprime Ltd., Cheadle Hulme, UK) coupled to a Eurovector CN elemental analyser (Eurovector SPA, Redavalle, Italy). Large samples received more oxygen, to improve combustion.

### Statistical analyses

All statistical analyses except ordination analyses were performed using SPSS version 23 (IBM) for PC. Ordinations were all analysed in Canoco for Windows version 4.5^44^. Only adult specimens were used for the species-level analyses, while analyses of total mite density and density of major groups included both adult and juvenile specimens. Density of total adult and juvenile mites (log-transformed) was analysed with univariate ANOVA, with treatment (control or warming) and site (mesic meadow, wet meadow and heath) as fixed factors. As the other data did not meet the assumption of normal distribution after transformation, the conservative nonparametric Mann-Whitney U test was used to analyse the effect of treatment on total mite density, density of mite groups and density of the 11 most common oribatid mite species across all plant communities. The Kruskal-Wallis test was used to analyse the effect of site on total soil mite density, density of the mite groups and density of the nine most common oribatid species. The uneven distribution of species among sites prevented a greater number of species being analysed. Plot-scale effect on total soil mite density and density of the mite groups was analysed with the Kruskal-Wallis test. The data were pooled within plots for the analyses on treatment and site effect, but not plot-scale effect. We are aware of the potential problem of pseudoreplication for the non-pooled data. The reason for including plot-scale effects was that others have shown that microarthropod abundance in polar regions can be heavily influenced by small-scale heterogeneity^45^. The Kruskal-Wallis test was used to analyse the effect of site on soil parameters (total N, total C, C/N ratio, moisture and pH in the organic and mineral soil layers). The Mann-Whitney U test was used to determine the effect of treatment on soil parameters within sites.

A constrained multivariate gradient analysis technique was used to examine the influence of the environmental variables (*i.e.* treatment, microsite and soil variables) on species composition and abundance of the most common mite species. DCA (Detrended Correspondence Analysis) showed that the data were suitable for a unimodal constrained ordination technique (*i.e.* had more than four standard deviation units for species turnover)^44^. We therefore decided to perform constrained CCA (Canonical Correspondence Analysis) with, in general, standard settings but with automatic selection of the environmental variables and with Monte Carlo unrestricted permutation tests with 1000 permutations to test the significance of the environmental constraints on CCA.

## Results

### Soil parameters

**Figure 1:**
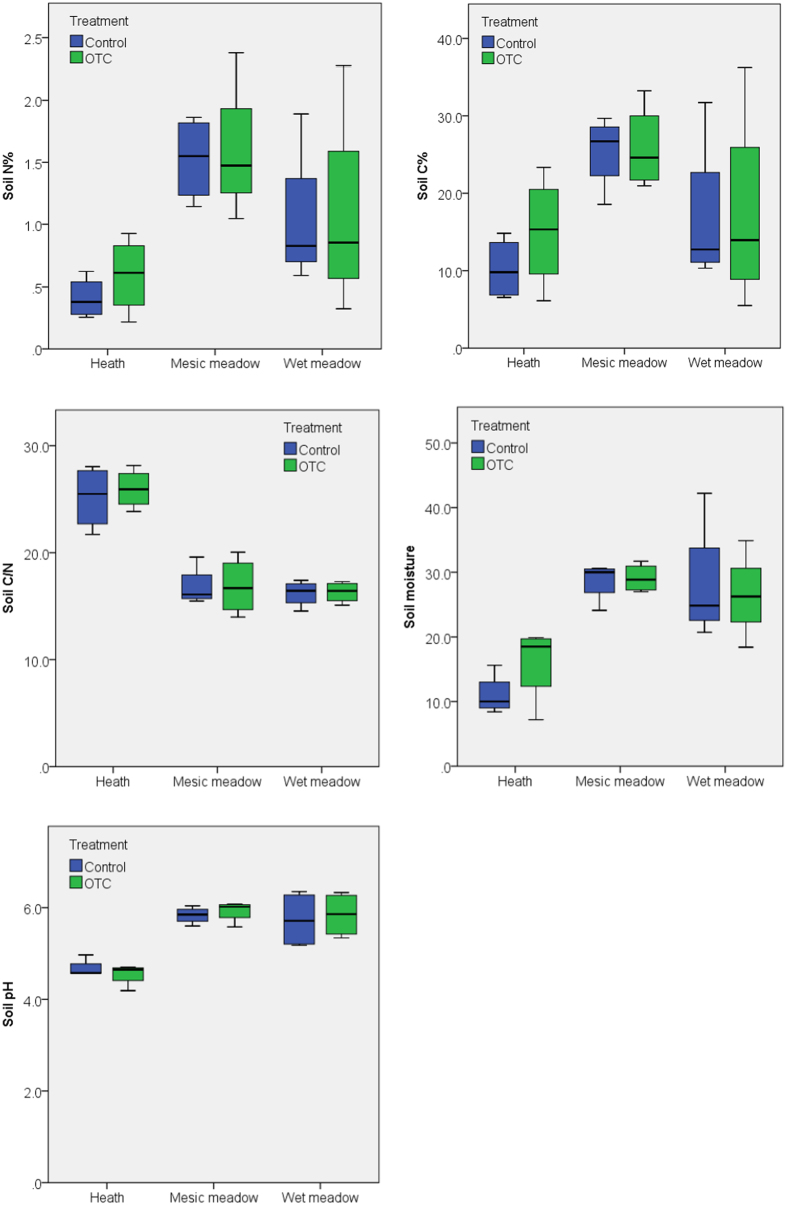
Box plots of soil parameters (total N, C, C/N ratio, soil moisture and pH in the organic soil layer) at the poor heath, mesic meadow and wet meadow study sites at Latnjajaure field station, subarctic Sweden. Treatments: control (CTR) and long-term warming (OTC). Boxplots show the 10^th^–90^th^ percentiles of the data; n = 4 for each site and treatment.

**Figure 2:**
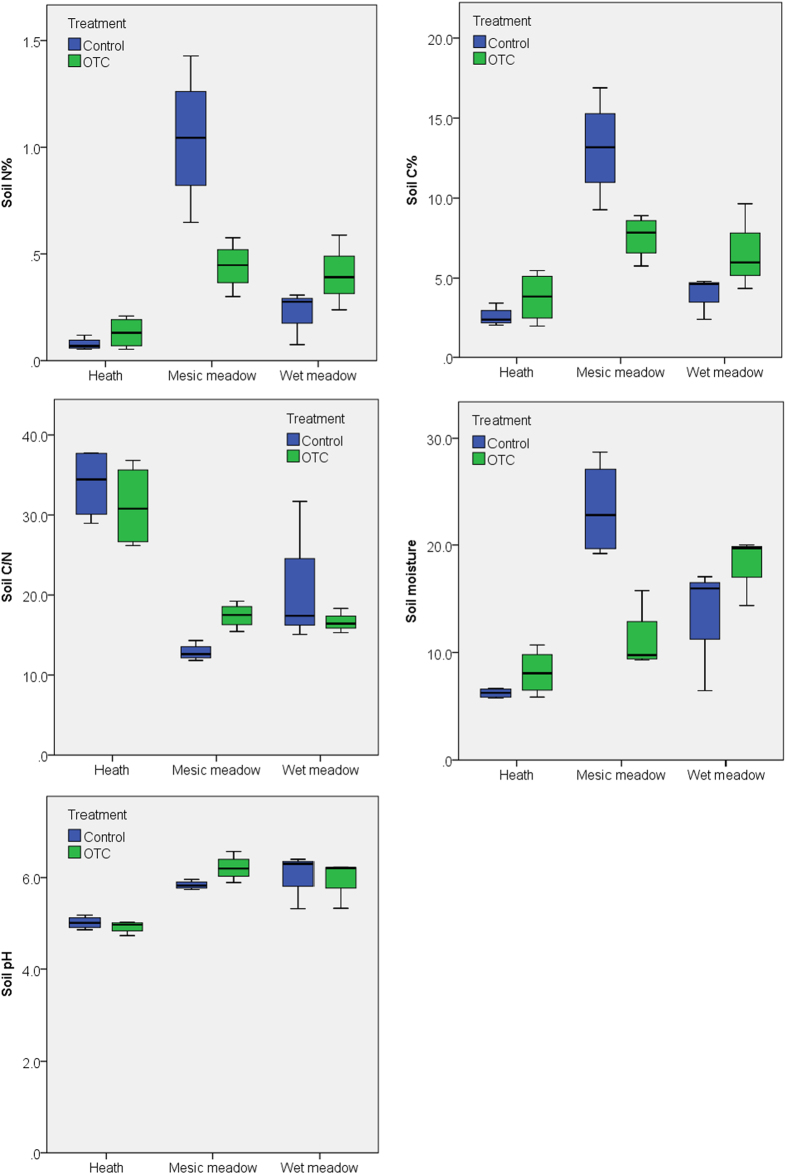
Box plots of soil parameters (total N, C, C/N ratio, soil moisture, and pH in the mineral soil layer) at the poor heath, mesic meadow and wet meadow study sites at Latnjajaure field station, subarctic Sweden. Treatments: control (CTR) and long-term warming (OTC). Boxplots show the 10^th^–90^th^ percentiles of the data; n = 4 for each site and treatment.

There was a significant site effect on the soil parameters measured in both the organic and mineral soil layers. These included total N (p = 0.001, p < 0.0001), C (p = 0.015, p = 0.001), C/N (p < 0.0001, p = 0.001), moisture (p < 0.0001, p = 0.004), and pH (p < 0.0001, p = 0.001). Soil N and C content ranged from low (poor heath) through intermediate (wet meadow) to high (mesic meadow) (Figs 1 and 2). The C/N ratio was highest in the poor heath and lower in the mesic and wet meadow (Figs 1 and 2). Soil moisture and pH were lowest in the poor heath and higher in the wet and mesic meadow (Figs 1 and 2). Long-term warming had no significant impact on soil parameters in the mineral soil layer in the heath and wet meadow (Fig. 2), but a significant negative effect on total N (p = 0.029), C (p = 0.029) and soil moisture (p = 0.029), a positive effect on C/N ratio (p = 0.029) and a near-significant positive impact on pH (p = 0.057) in the mineral soil layer of the meadow (Fig. 2). However, there were no significant effects on the soil parameters in the organic soil layer in the meadow, wet meadow or heath (Fig. 1). Plot had no impact on any of the soil parameters in the organic or mineral soil layers.

### Dominance structure of mite groups

**Figure 3:**
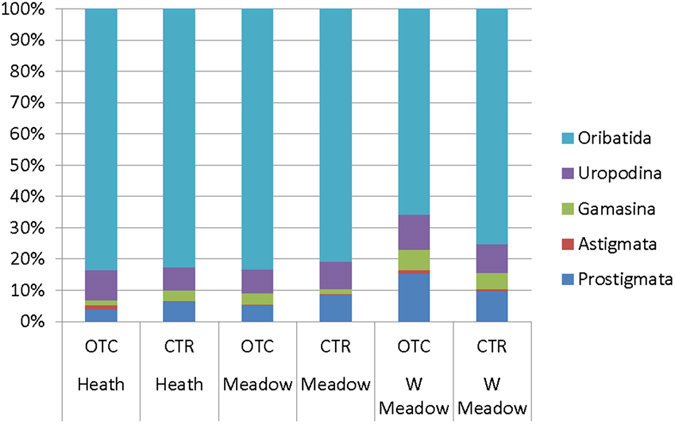
Relative dominance of different mite groups (Prostigmata, Astigmata, Gamasina, Uropodina and Oribatida) in soil at the poor heath, mesic meadow and wet meadow sites at Latnjajaure field station, subarctic Sweden. Treatments: control (CTR) and long-term warming (OTC).

We found that while dominance structure differed between groups, the distribution pattern within groups was fairly similar for all three sites (Fig. 3). There was a clear dominance structure among groups, with saprophagous mites dominating at all sites. Oribatida had a relative dominance of more than 80% at the mesic meadow and heath sites and over 65% at the wet meadow site (Fig. 3). Uropodina (7–11%) and Prostigmata (4–15%) were also common at all sites, while Astigmata (0.3–1.3%) and predatory Gamasina (1.6–6.7%) were much less frequent (Fig. 3).

### Soil mite abundance

**Figure 4:**
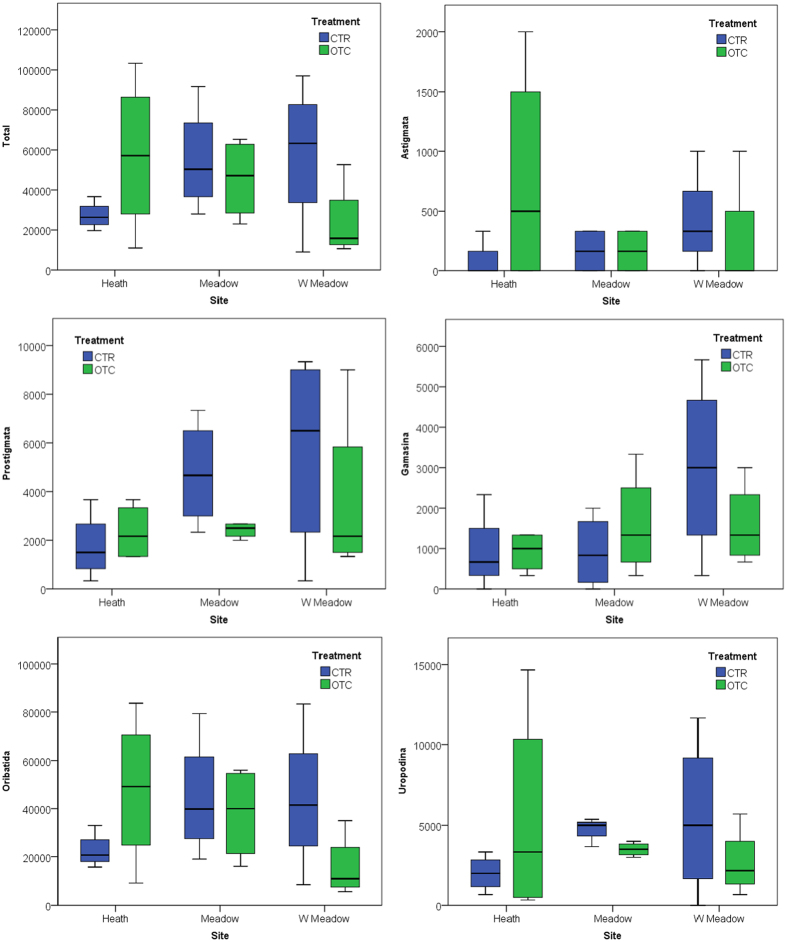
Box plots of mean total mite abundance (ind. m^−2^) and total abundance (ind. m^−2^) of the Prostigmata, Astigmata, Gamasina, Uropodina and Oribatida groups of soil mites at the poor heath, mesic meadow and wet meadow study sites at Latnjajaure field station, subarctic Sweden. Treatments: control (CTR) and long-term warming (OTC). Boxplots show the 10^th^–90^th^ percentiles of the data; n = 4 for each site and treatment.

**Figure 5:**
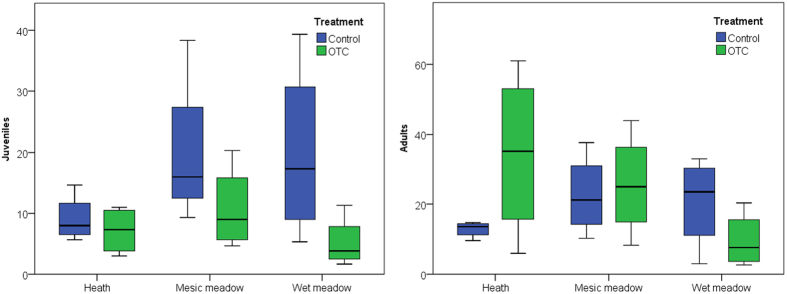
Box plots of mean abundance (×10^3^ ind. m^−2^) of total juvenile and adult oribatid mites at the poor heath, mesic meadow and wet meadow study sites at Latnjajaure field station, subarctic Sweden. Treatments: control (CTR) and long-term warming (OTC). Boxplots show the 10^th^–90^th^ percentiles of the data; n = 4 for each site and treatment.

The 19 years (mesic meadow and poor heath) or 21 years (wet meadow) of experimental warming had no significant effect on density of any of the major mite groups: Prostigmata (p = 0.378), Astigmata (p = 1.00), Gamasina (p = 0.887), Uropodina (p = 0.713) and Oribatida (p = 0.713), or on total mite density (p = 0.671) at any of the three sites (Fig. 4). However, there was a significant treatment effect on juvenile oribatids, the number of which decreased under long-term warming, but not on adult mites (Table S1, Fig. 5). While not significant, long-term warming tended to have a negative effect on Prostigmata (p = 0.11) and Uropodina (p = 0.057) in the mesic meadow (Fig. 4). Similarly, there was no significant site effect on density of the major mite groups: Prostigmata (p = 0.215), Astigmata (p = 0.918), Gamasina (p = 0.191), Uropodina (p = 0.371) and Oribatida (p = 0.385), or on total mite density (p = 0.553). The importance of spatial heterogeneity was underscored by the fact that plot-scale heterogeneity was the only factor with a significant effect on mite density and was thus an important controlling factor. It had significant effects on the density of Gamasina (p = 0.024), Uropodina (p = 0.048) and Oribatida (p = 0.01) and on total density (p = 0.001), and close to significant effects on the density of Prostigmata (p = 0.099) and Astigmata (p = 0.080).

### Species-level responses

A total of 59 species of oribatid mites (adult specimens) were found in this study (Table S2). On species level, there was no treatment effect on any of the most common species: *Belba compta* (p = 0.905), *Dissorhina ornata* (p = 0.181), *Eobrachychthonius latior* (p = 0.073), *Liochthonius strenzkei* (p = 0.613), *Neonothrus humicolus* (p = 0.686), *Oppiella acuminata* (p = 1.00), *Oppiella neerlandica* (p = 0.799), *Oppiella unicarinata* (p = 0.40), *Oribatula tibialis* (p = 1.00), *Platynothrus peltifer* (p = 0.093) and *Tectocepheus velatus velatus* (p = 0.156) (Fig. S1). There was a site effect on one species, *Oppiella neerlandica* (p = 0.030), but not on *Belba compta* (p = 0.542), *Dissorhina ornata* (p = 0.073), *Eobrachychthonius latior* (p = 0.396), *Liochthonius strenzkei* (p = 0.689), *Oppiella unicarinata* (p = 0.846), *Oribatula tibialis* (p = 0.296), *Platynothrus peltifer* (p = 0.0968) and *Tectocepheus velatus velatus* (p = 0.20) (Fig. S1).

### Effect of environmental variables on composition and abundance of mites

**Figure 6:**
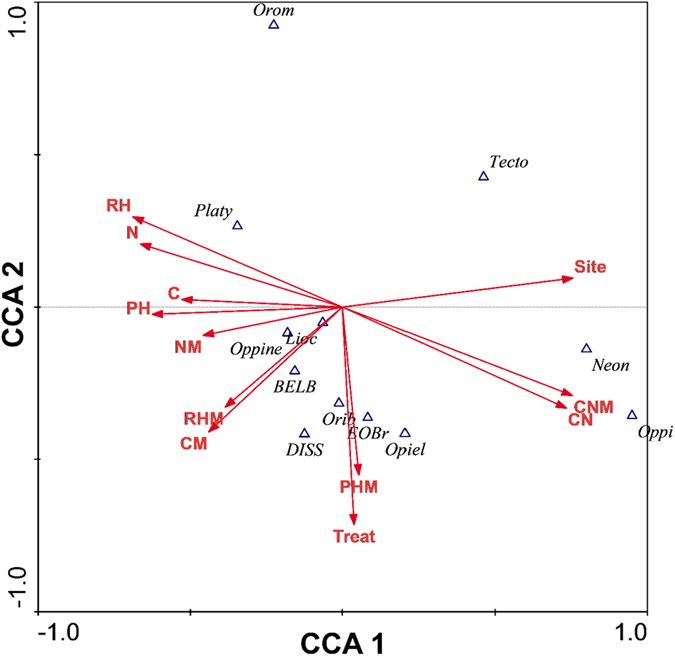
Plot of constrained Canonical Correspondence Analysis (CCA) on abundance change in mite species/groups after 19 (mesic meadow and poor heath) and 21 (wet meadow) years of experimental perturbations. CCA1 and CCA2 explain 70.5% of the variation (see Table S2). Environmental variables: N (N% in organic soil layer), C (C% in organic soil layer), CN (C/N ratio in organic soil layer), RH (moisture% in organic soil layer), PH (pH in organic soil layer), NM (N% in mineral soil layer), CM (C% in mineral soil layer), CNM (C/N in mineral soil layer), RHM (moisture% in mineral soil layer), PHM (pH in mineral soil layer). Species abbreviations: BELB = *Belba compta*, DISS = *Dissorhina ornata*, EOBr = *Eobrachychthonius latior*, Lioc = *Liochthonius strenzkei*, Neon = *Neonothrus humicolus*, Oppi = *Oppiella acuminata*, Oppine = *Oppiella neerlandica*, Opiel = *Oppiella unicarinata*, Orib = *Oribatula tibialis*, Orom = *Oromurcia cf. sudetica*, Platy = *Platynothrus peltifer*, Tecto = *Tectocepheus velatus velatus*.

The first three axes of the constrained CCA explained 88.3% of the variation (Table S3), and the permutation tests showed that warming (treatment), C, C/N and moisture in the organic soil, and C in the mineral soil, had significant influence on mites (treatment p = 0.005, F = 3.25; C in the organic soil p = 0.02, F = 2.69; C/N in the organic soil p = 0.0010, F = 4.92; moisture in the organic soil p = 0.038, F = 2.19; C in the mineral soil p = 0.017, F = 2.49). Other variables did not have any significant effects (Fig. 6), although two environmental variables had p < 0.10: pH in the organic soil (p = 0.083, F = 1.92) and C/N ratio in the mineral soil (p = 0.062, F = 2.00). The species-environmental correlations in the first three axes were high (0.94; 0.941, 0.933), and the cumulative percentage variance showed that the species-environment relationship was high for all axes (34.2%, 63.6%, 79.6%, respectively, for the three CCA axes) (Table S3).

The first CCA axis (CCA1) showed a high correlation with C/N ratio (in the organic soil) and site on the right side, associated with high abundance of *O. acuminata* (Oppi) and *N. humicolus* (Neon). On the left side of CCA1, moisture and C in the organic soil and C in the mineral soil dominated, together with a high number of other soil variables (Fig. 6). The second axis (CCA2) showed high correlation with the warming treatment and many species. *Dissorhina ornata* (DISS), *O. tibialis* (Orib), *E. latior* (EOBr) and *O. unicarinata* (Opiel)) were situated and clustered near treatment in CCA2, indicating higher abundance with the warming treatment. *Oromurcia cf. sudetica* (Orom), *P. peltifer* (Platy), and *T. velatus velatus* (Tecto) seemed to decrease in abundance with the warming treatment (Fig. 6).

## Discussion

This study revealed that two decades of warming had contrasting effects on soils across alpine tundra plant communities. In the mineral soil layer of the mesic meadow, the long-term warming caused a decrease in total nitrogen and carbon, a positive effect on C/N ratio and a near-significant positive effect on pH. However, no effects were found on the organic soil layer of the mesic meadow or in the mineral and organic soils layers of the wet meadow and poor heath communities. This is similar to the long-term nutrient enhancement study that found a loss of carbon and nitrogen in the mineral, but not organic soil layers^9^. Decomposition increasing more than plant production causing a net loss of carbon^9^. Thus, the impact of global warming may cause different responses in soils on local scale. This has implications for modelling global carbon balance, as Arctic tundra ecosystems contain a vast carbon pool. To be realistic, models will thus need to include local-scale heterogeneity of potential impacts of global warming on carbon balance. Previous studies have shown that 20 years of fertilisation in Arctic tundra caused a decrease in carbon and nitrogen storage^9^, while 20 years of warming did not change total soil carbon or nitrogen^4^. Furthermore, a recent study showed that global warming is likely to cause substantial carbon loss in high latitude areas, the effect of warming depending on the initial soil carbon stock^46^. The OTCs have also been shown to warm soil by 1.4 °C^14^ and to cause increased drought at both Antarctic and Arctic sites^15^. In this study we observed a decrease in soil moisture in the mineral soil layer in the mesic meadow community, but this was not the case in the wet meadow or poor heath community or in the organic soil layer at any site. Thus, the impact on soil moisture most likely depends on the local heterogeneity of soils.

We had expected to find the highest soil moisture in the wet meadow community, but this was not the case. This may have been due to the unusually warm summer of 2013, which caused early melt of snowbeds in the surrounding valley. This affected the duration of meltwater feed into the wet meadow, causing a drier than usual environment in the peak vegetation period in late July and early August (Ulf Molau, personal communication 2016). At the time of the sampling, waterproof footwear was not necessary, which was unusual. If climate change causes higher early summer temperatures, this would potentially impose major changes in plant communities in the valley, decreasing snowbed communities and wet plant communities and favouring more mesic plant communities, as the soils would become drier.

The lack of effect of two decades of warming on soil moisture, carbon, nitrogen, C/N ratio and pH of the organic soil layers across the three sites included in this study may explain why we found no effect of long-term warming on total mite density, any major mite group or the most common oribatid species. Organic material and soil moisture are generally important for soil fauna^18^,^27^,^47^. This was also the case in the present study, where multivariate analyses showed that carbon, C/N ratio and moisture all had a significant influence on mites. However, multivariate analyses showed a significant treatment effect on the most common species as a group. This indicates that while long-term warming may not have had any effect on total mite community or any of the most common individual species, the most common species were affected as a group. Organic soil layers are thin at the sites^48^ and, as mites also occur in the mineral soil layer, the lack of response in the mesic meadow, which experienced a loss of carbon, nitrogen and moisture in the mineral soil layer, is difficult to explain. Similarly, the lack of site effect on total mite density was unexpected as there was a significant site effect on all soil parameters. In particular, we expected to find lower mite densities in the poor heath, as it had lower carbon, nitrogen and moisture levels than the mesic and wet meadow communities.

We found a significant negative effect of long-term warming on juvenile oribatids, but not on adult mites. This is line with previous research suggesting that mites tend to be less sensitive than Collembola to moisture levels^28^,^29^, but that mite nymphs may still be vulnerable^30^,^31^. Another potential explanation may be that the experimental plots lacked barriers within the soil, so adult soil mites could have re-colonised the warmed plots even when the conditions may have been less favourable for juveniles. A third potential explanation may be that due to the warming, the mites in the experimental plots perhaps reproduced and hatched earlier and also grew faster than those in the control plots, so at the time of sampling many of them had already developed into adults, decreasing the number of juveniles in the samples.

Plot-scale heterogeneity was an important factor for soil mites. As commonly reported in other alpine areas^49^, the soil depth in the sampling sites was generally both thin and highly variable, depending on microhabitat^48^. The organic layer only reached a depth of 1–10 cm, depending on site, and was sometimes almost non-existent in the poor heath community. This was reflected during sampling, when we frequently had to try for new spots as the soil core sampler hit stones in the soil. This most likely affected the spatial distribution of mites. A similar plot-scale effect was found previously in a study on Collembola at the same sites^32^. Small-scale heterogeneity could thus have an important buffering effect on soil fauna^32^, in a similar way as boulders in high alpine areas can buffer species from extreme heat events^50^. Studies on mountain-top boulder fields have shown that they can provide microhabitat temperature buffering for extreme events (heat waves) over short distances, and that they have probably functioned as important refugia during historical climate fluctuations^50^. This is important, as climate change will most likely increase the variability and frequency of extreme events^51^,^52^, and thus a constant level of warming is not a very realistic scenario for the future. However, at present there are no long-term experiments applying different warming scenarios on plant communities and only a few multi-year experiments in alpine and Arctic areas^53^,^54^,^55^,^56^. In the future, a high level of soil heterogeneity could thus potentially provide refugia from where adult stages of microarthropods can recolonise areas where juvenile stages have been negatively affected by climate change/extreme events.

A similar, but short-term, study to this found no effects of four years of experimental warming on oribatid mites^15^. However, the life cycle of oribatid mites often spans several years^39^, and this, in combination with high weather variability between years, may have masked the effects of the limited experimental warming applied in that study^15^,^25^. This interaction between between-year variability and experimental warming effect has previously been demonstrated in a short-term study which found that the warming effect on soil fauna depended on whether the summer was relatively warm and dry, or cool and wet^57^. The warming had positive effects on mesofaunal biomass and diversity in the cool and wet year, a negative effect in the drier zone and a positive effect in the moister zone in the warmer year^57^. Thus, it could be predicted that at sites with higher soil moisture levels, microarthropods would show positive responses to long-term warming, while at sites with lower soil moisture levels microarthropods would show negative responses. In the present study, the sampling year 2013 was warm and dry, which could therefore have been predicted to have a negative effect on the dry site and a more positive effect on the sites with higher soil moisture. However, while not significant, we found the opposite pattern, with the warming tending to have a positive effect on mite density/abundance at the dry site and a negative effect at the sites with higher soil moisture. A potential explanation for this may be the non-significant, but slightly higher, nitrogen, carbon and moisture levels in the organic and mineral layers of the warmed plots in the heath. Thus, even a small increase in the dry, nutrient and carbon-poor organic and mineral soil layers may have a positive effect on mite densities.

We also expected a site effect on mites due to differences in the natural nutrient gradient between sites. A possible explanation for the lack of treatment and site effects on mites may be the limited sample size, with only four replicates per treatment. The only significant effect we observed was on one species, *O. neerlandica*, and at plot-scale on Gamasina, Uropodina, Oribatida and total density, most likely reflecting the high heterogeneity of the soils in the Latnjavagge valley.

To conclude, we showed that the impact of two decades of experimental warming on carbon, nitrogen and soil moisture in the mineral layer differed among local plant communities, causing a loss in a mesic alpine meadow, but not in nearby wet meadow, or nutrient-poor dry heath. These results have important implications for modelling global carbon balance, as Arctic soils contain a large pool of carbon. The results can help improve understanding of the potential impact of global warming on carbon balance in heterogeneous soils, e.g. by showing that responses may be highly variable both at larger^46^ and local scale. The long-term warming had no effect on total mite abundance or on any of the most common oribatid species. However, global warming had differing impacts on juvenile and adult stages of oribatids, with juvenile stages being more vulnerable. Furthermore, the importance of small-scale variance indicates that local soil heterogeneity may play an important role in providing refugia for soil microarthropods under future climate change/extreme events.

## Additional Information

**How to cite this article**: Alatalo, J. M. *et al*. Impacts of twenty years of experimental warming on soil carbon, nitrogen, moisture and soil mites across alpine/subarctic tundra communities. *Sci. Rep.* **7**, 44489; doi: 10.1038/srep44489 (2017).

**Publisher's note:** Springer Nature remains neutral with regard to jurisdictional claims in published maps and institutional affiliations.

## Supplementary Material

Supplementary Material

Supplementary Dataset 1
