# Supplementary material for: Impacts of twenty years of experimental warming on soil carbon, nitrogen, moisture and soil mites across alpine/subarctic tundra communities
Source: Sci Rep. 2017 Mar 15;7:44489. doi: 10.1038/srep44489 (PMC5353735; doi:10.1038/srep44489)
Supplement: Supplementary Material [file srep44489-s1.pdf]

Supplementary material for “Impacts of twenty years of experimental warming on soil carbon, nitrogen, moisture and soil mites across alpine/subarctic tundra communities”

Authors: Juha M. Alatalo[1*], Annika K. Jägerbrand[2], Jaanis Juhanson[3], Anders Michelsen[4,5], and Peter Ľuptáčik[6]

[1]Department of Biological and Environmental Sciences, College of Arts and Sciences, Qatar University, P.O. Box 2713, Doha, Qatar; [2]Calluna AB, Hästholmsvägen 28, 131 30 Nacka, Sweden; [3]Swedish University of Agricultural Sciences, Dept. of Forest Mycology and Plant Pathology, P.O Box 7026, SE-75007 Uppsala, Sweden; [4]Terrestrial Ecology Section, Department of Biology, University of Copenhagen, Copenhagen, Denmark; [5]Center for Permafrost (CENPERM), University of Copenhagen, Øster Voldgade 10, DK-1350 Copenhagen K, Denmark; [6]Institute of Biology and Ecology, Faculty of Science, P. J. Šafárik University in Košice, Šrobárova 2, 041 54 Košice, Slovakia.

*Corresponding author: jalatalo@qu.edu.qa

Supplementary Figure S1, p.2-3

Supplementary Tables S1-S3, p. 4-6.

Figure S1. Box plots of mean abundance (ind.$^{-3}$ m^{2}) of the 11 most common oribatid mite species at the poor heath, mesic meadow and wet meadow study sites at Latnjajaure field station, subarctic Sweden. Treatments: control (CTR) and long-term warming (OTC). Boxplots show the 10th-90th percentiles of the data; n = 4 for each site and treatment.

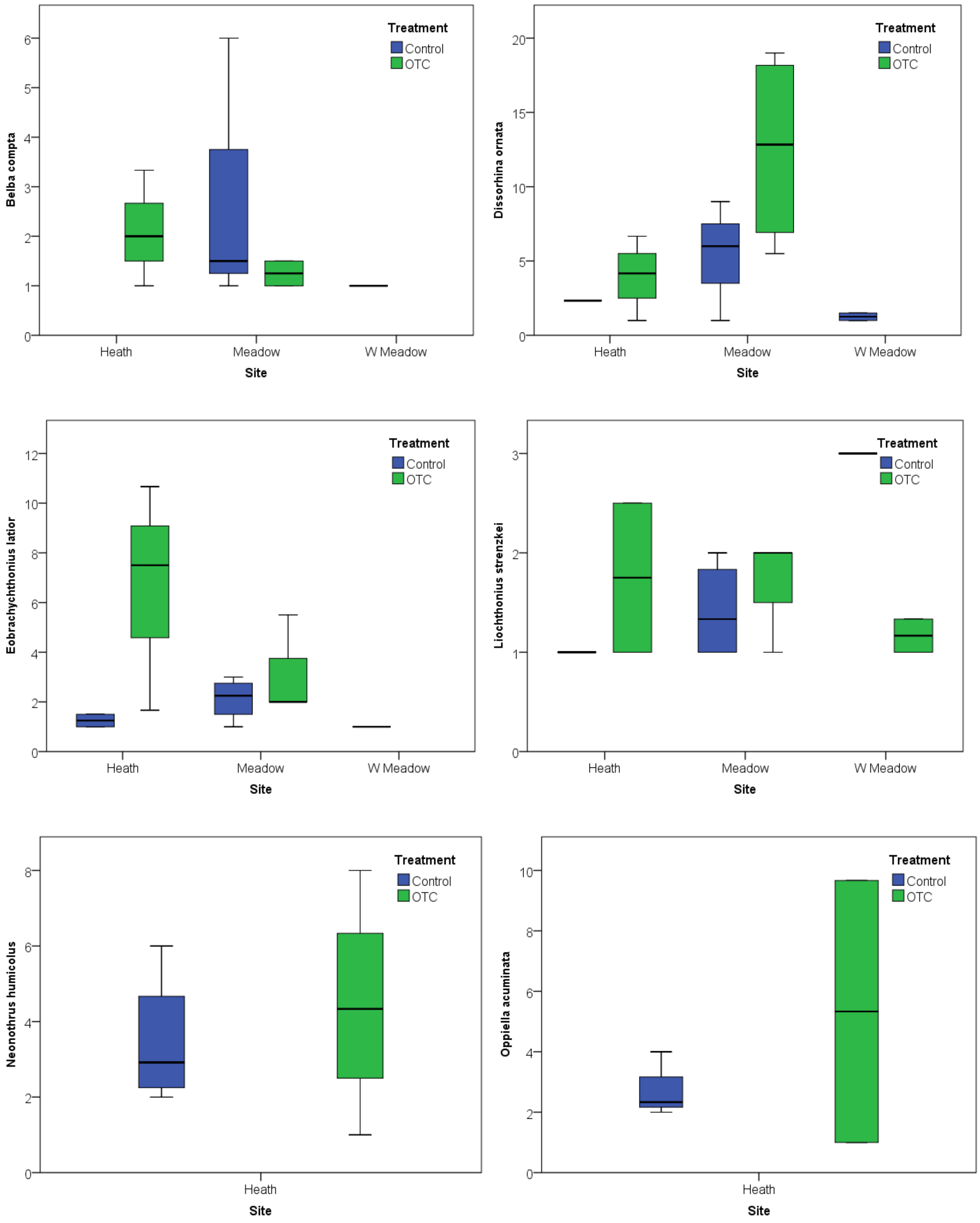

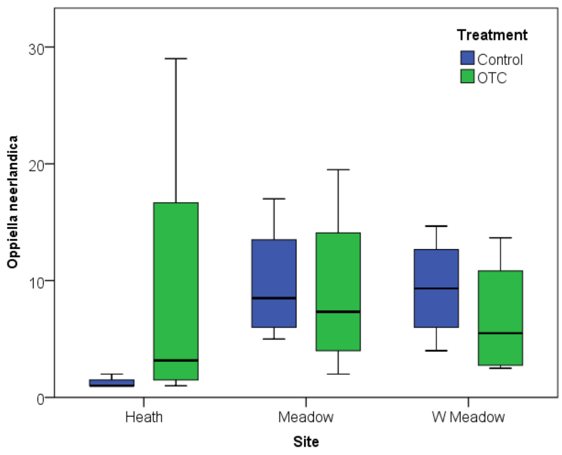
Treatment
Control
OTC
Oppiella neerlandica
0
10
20
30
Heath
Meadow
W Meadow
Site

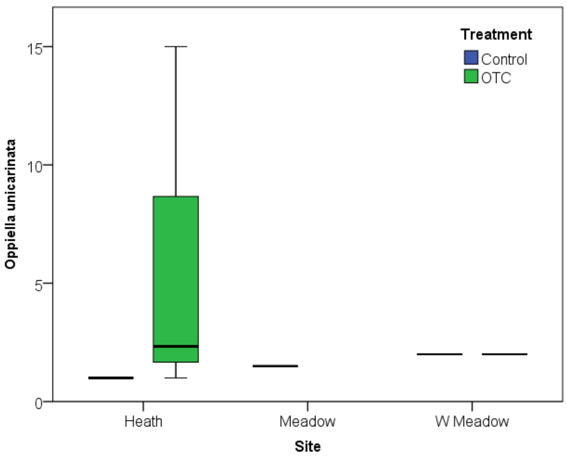
Treatment
Control
OTC
Oppiella unicarinata
0
5
10
15
Heath
Meadow
W Meadow
Site

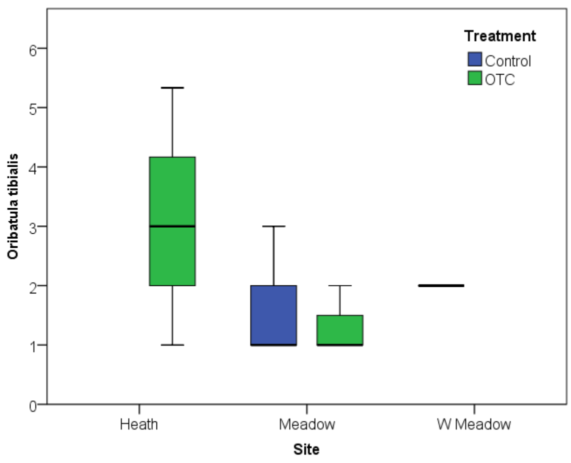
Treatment
Control
OTC
Oribatula tibialis
0
1
2
3
4
5
6
Heath
Meadow
W Meadow
Site

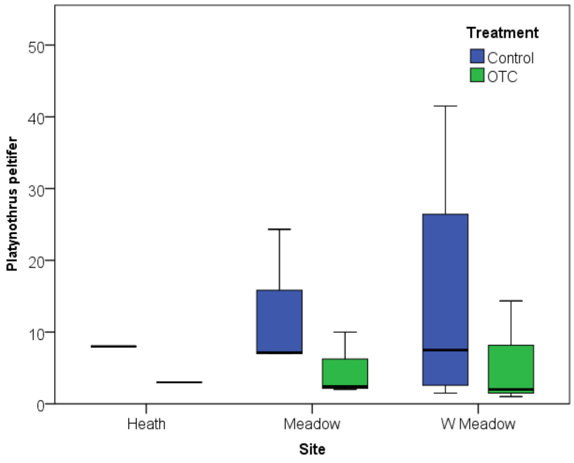
Treatment
Control
OTC
Platynothrus peltifer
0
10
20
30
40
50
Heath
Meadow
W Meadow
Site

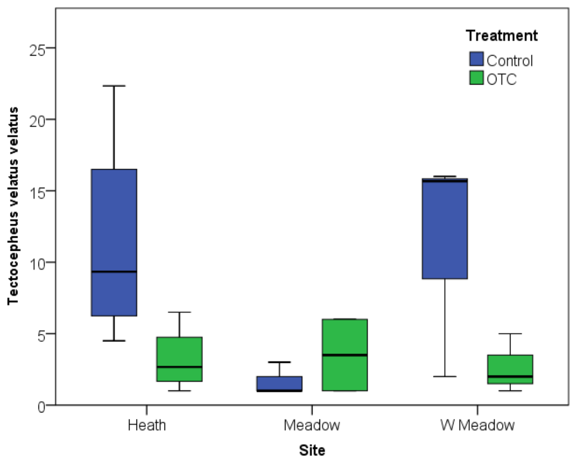
Treatment
Control
OTC
Tectocepheus velatus velatus
0
5
10
15
20
25
Heath
Meadow
W Meadow
Site

Table S1. Result of univariate ANOVAs testing the effects of treatment (19 and 21 years of experimental warming) and site (rich alpine meadow, wet alpine meadow and poor alpine heath) on abundance of juvenile and adult soil mites. df = degrees of freedom, F = F-statistics, Sig. = significance level

Source	Type III Sum of Squares	df	Mean Sq.	F	Sig.
**Juvenile mites**					
Corrected model	6.000[a]	5	1.200	2.710	.054
Intercept	116.155	1	116.155	262.305	.000
Treatment	3.486	1	3.486	7.872	.012
Site	1.398	2	.699	1.578	.234
Treatment * Site	1.116	2	.558	1.260	.308
Error	7.971	18	.443		
Total	130.125	24			
Corrected total	13.970	23			

a. R Sq. = .429 (Adjusted R Sq. = .271)

Source	Type III Sum of Squares	df	Mean Sq.	F	Sig.
**Adult mites**					
Corrected model	1593.81[a]	5	318.696	1.642	.200
Intercept	10528.074	1	10528.074	54.251	.000
Treatment	118.519	1	118.519	.611	.445
Site	401.593	2	200.796	1.035	.376
Treatment * Site	1073.370	2	536.685	2.766	.090
Error	3493.111	18	194.062		
Total	15614.667	24			
Corrected total	5086.593	23			

a. R Sq. = .313 (Adjusted R Sq. = .123)

Table S2. List of mite species (adult specimens) found at Latnjajaure.

Achipteria nitens	Heminothrus longisetosus	Oribatula tibialis
Banksinoma lanceolata	Chamobates borealis	Oromurcia cf. sudetica
Belba compta	Chamobates spinosus	Platynothrus aff. peltifer
Brachychochthonius cf. immaculatus	Kunstidamaeus nidicola	Platynothrus peltifer
Camisia biurus	Liochthonius cf. leptaleus	Quadroppia quadricarinata
Camisia biverrucata	Liochthonius neglectus	Suctobelba acutidens
Camisia horrida	Liochthonius sp.	Suctobelba regia
Carabodes labyrinthicus	Liochthonius strenzkei	Suctobelba trigona
Ceratizetes macromediocris	Melanozetes mollicomus	Suctobelbella acutidens
Ceratoppia shaerica	Mycobates sarekensis	Suctobelbella arcana
Ceratoppia sphaerica	Neonothrus humicolus	Suctobelbella cf. arcana
Ceratozetes parvulus	Nothrus borussicus	Suctobelbella cf. nasalis
Ceratozetes thienemanni	Nothrus silvestris	Suctobelbella cf. sarekensis
Dissorhina ornata	Oppiella acuminata	Suctobelbella sarekensis
Edwardzetes edwardsi	Oppiella aff. marginedentata	Suctobelbella subcornigera
Eobrachychthonius latior	Oppiella neerlandica	Tectocepheus velatus sarekensis
Eremaeus hepaticus	Oppiella nova	Tectocepheus velatus velatus
Eupelops plicatus	Oppiella sp. (hygrophila)	Tegoribates latirostris
Fuscozetes setosus	Oppiella subpectinata	Trichoribates sp.
Fuscozetes sp.	Oppiella unicarinata	

Table S3. Result of Canonical Correspondence Analysis (CCA) on abundance change of the most common mite species after 19 (mesic meadow and poor heath) and 21 (wet meadow) years of experimental perturbations.

Axes	1	2	3	4	Total inertia
Eigenvalues:	0.379	0.326	0.178	0.099	1.478
Species-environment correlations:	0.94	0.941	0.933	0.805	
Cumulative percentage variance					
of species data:	25.7	47.7	59.8	66.5	
of species-environment relation:	34.2	63.6	79.6	88.5	
Sum of all eigenvalues					1.478
Sum of all canonical eigenvalues					1.11
